# A comparative probabilistic analysis of human and chimpanzee rotator cuff functional capacity

**DOI:** 10.1111/joa.13882

**Published:** 2023-04-25

**Authors:** Kathleen F. E. MacLean, Joseph E. Langenderfer, Clark R. Dickerson

**Affiliations:** ^1^ Department of Kinesiology University of Waterloo Waterloo Ontario Canada; ^2^ College of Science & Engineering, Central Michigan University Mount Pleasant Michigan USA

**Keywords:** biomechanics, glenoid inclination, glenoid stability, human evolution, muscle attachments, rotator cuff pathology, shoulder function, stochastic modeling

## Abstract

Computational musculoskeletal modeling represents a valuable approach to examining biological systems in physical anthropology. Probabilistic modeling builds on computational musculoskeletal models by associating mathematical distributions of specific musculoskeletal features within known ranges of biological variability with functional outcomes. The purpose of this study was to determine if overlap in rotator cuff muscle force predictions would occur between species during the performance of an evolutionarily relevant horizontal bimanual arm suspension task. This necessitated creating novel probabilistic models of the human and chimpanzee glenohumeral joint through augmentation of previously published deterministic models. Glenohumeral musculoskeletal features of anthropological interest were probabilistically modeled to produce distributions of predicted human and chimpanzee rotator cuff muscle force that were representative of the specific anatomical manipulations. Musculoskeletal features modeled probabilistically included rotator cuff origins and deltoid insertion, glenoid inclination, and joint stability. Predicted human rotator cuff muscle force distributions were mostly limited to alternating between infraspinatus and teres minor, with both 100% and 0% muscle force predicted for both muscles. The chimpanzee model predicted low‐to‐moderate muscle force across all rotator cuff muscles. Rotator cuff muscle force predictions were most sensitive to changes of muscle origins and insertions. Results indicate that functional rotator cuff overlap is unlikely between chimpanzees and humans without greater modifications of the glenohumeral musculoskeletal phenotypes. The results also highlight the low efficacy of the human upper extremity in overhead, weight‐bearing tasks, and propensity for rotator cuff injury.

## Graphical abstract

Human and chimpanzee rotator cuff functional overlap was explored using probabilistic musculoskeletal modeling simulations. Regardless of probabilistic modification to anthropologically significant musculoskeletal shoulder anatomy, humans were predicted to overload infraspinatus and teres minor in the computational simulations. The lack of functional species convergence in rotator cuff action and human‐specific infraspinatus overload reveals an anatomical evolutionary pathway for rotator cuff pathology in humans

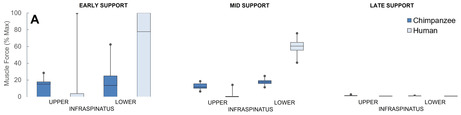

## 1 INTRODUCTION

Morphological comparisons, which are common in physical anthropology, have led to major breakthroughs in understanding functional adaptations to the musculoskeletal form. Much of the present knowledge regarding human evolution arose from comparative morphology and morphometrics by comparing boney features between primate species and fossils to enable meaningful associations of the physical capabilities with modern function (Larson, 2018). However, this method of evaluating and analyzing morphology through an “itemized” comparative approach is challenging (Lovejoy et al., 1999), as it is difficult to determine which isolated features are most important for locomotion and most related to each other (Stern & Susman, 1991). Further, certain features vary markedly even within species, and the influence of this plasticity on and contribution to functional ability is unknown (Collard & Wood, 2000). Since the entire organism acts as a system, slight variations through development and growth accumulate, and modify other parts of the system.

Computer models that can simulate small morphological changes provide key insights into how specific traits may affect physical ability and joint function within a musculoskeletal system (Chopp‐Hurley et al., 2016; Hutchinson, 2012). The complexity of the shoulder, and scapular morphology in particular, complicates reaching conclusions regarding the development of species‐specific characteristics and defeats traditional morphological comparison methods (Young, 2003). While morphological traits can be similar between specific primate species, these species may not share locomotor and functional behaviors. The modern human scapula is believed to most closely resemble the orangutan scapula (Oxnard, 1967, 1977), despite these two species having the most divergent functional behaviors of the great apes. Therefore, similar bony morphology in two different species may potentially yield different behaviors due to changes in other components of the musculoskeletal system. This duplicity may create different roles for similar physiological or morphological traits (Lauder, 1995). It also may explain many of the current divergent views in the literature around the association between scapular morphology and shoulder function and evolution, when morphological comparisons are employed (Almecija, 2016; Melillo, 2015; Young et al., 2015). Therefore, individual or select morphological features should not be considered in isolation, but rather must be contextualized as part of a complex plastic system. Computational musculoskeletal modeling can simulate these systems, concurrently with functional behaviors, and help to identify candidate traits that are integral to each species' physical ability. This may in turn help to settle persistent debates regarding the evolutionary relationship between form and function in primates and humans.

Probabilistic or stochastic computational musculoskeletal modeling further enables novel insights into the manifestation of functional variability within populations and between species. For instance, a single deterministic biomechanical computational model representing a single anatomical condition will output single representative values for specific inputs and parameters of the computational model. Stochastic or probabilistic models can randomly vary or perturb specific anatomical inputs and parameters within a single biomechanical computational model to produce a distribution of output values. Deterministic computational models can incorporate known or suspected musculoskeletal differences in a variety of tasks, postures, and morphologies to allow crucial simulation of injury mechanisms, as they are often multifactorial and dependent on both kinematics and morphological variations (Chopp‐Hurley et al., 2016). However, deterministic models lack consideration of the breadth of variability of the determinants of injury and performance within a population (Langenderfer et al., 2006). Instead, the stochastic approach provides mechanisms for capturing the effects of physiological variation between individuals, populations, and species. Small iterative changes to the parameters within musculoskeletal models using probabilistic methods, much like in biological organisms, can result in pronounced changes in musculoskeletal function (Chopp‐Hurley et al., 2014; Hutchinson, 2012; Larson, 1998; Young et al., 2015). To fully understand the influence of evolutionarily relevant physical features on the distribution of modern human function, the full consequences of species variation on those features should be explored through a probabilistic computational approach.

The modern human shoulder is susceptible to rotator cuff injury in overhead and weight‐bearing postures due to evolutionary musculoskeletal organization (Chopp et al., 2010; Cote & Bement, 2010; Lewis et al., 2001; Stauber, 2004). Rates of rotator cuff disease are very common in modern humans compared with other primate species, despite a habitually non‐weight‐bearing upper extremity (Roberts, 2008). In humans who actively engage in climbing, upward of a third present with rotator cuff injuries (Rooks, 1997). Higher rates of rotator cuff disease have been noted in humans over the age of 60 (Bartolozzi et al., 1994). This, despite most humans not habitually engaging in repetitive, weight‐bearing, overhead behaviors like many of their primate relatives. The human pathological predisposition is likely rooted in specific musculoskeletal organizational differences between humans and other closely related species, such as chimpanzees (Lewis et al., 2001; Roberts, 2008; Voisin et al., 2014).

As physical form and function have a complex but clear association, there are numerous musculoskeletal features that have been proposed as evolutionary modulators of overhead shoulder function in humans and other closely related primates. Among those that may affect rotator cuff muscle activity are changes to rotator cuff and deltoid muscle footprints, orientation of the glenohumeral joint, and the multifactorial stability of the glenohumeral joint. Changes to the shape of bony attachment sites are analogous to changes in muscle footprints (Larson, 1995), such as the length, width, and depth of the infraspinatus and supraspinatus fossa and deltoid tuberosity (Craik et al., 2014). Muscle attachment modifications can change the direction of muscle lines of action, which will also change muscle coordination around the joint. Humans have a wider and shorter scapular body shape relative to chimpanzees, modifying the lines of actions of all rotator cuff muscles around the glenohumeral joint and muscle force sharing. Glenohumeral superior–inferior orientation and scapular spine orientation are closely associated and can modify the optimized range of motion of a joint and muscle lines of action around the joint (Larson, 1995). Humans have a less superiorly orientated glenoid (Lewis et al., 2001), reducing the capacity for superior, overhead behaviors such as arborealism. Glenohumeral stability is multifactorial. Among the factors that affect glenohumeral stability are articular version, labrum, joint pressure, adhesion mechanics, proprioception, ligaments, and muscle activity (Cole et al., 2007; Veeger & van der Helm, 2007). As the specific contribution of each factor is unknown, stability can be represented as a mathematical composite ratio of shearing joint force tolerance relative to compressive joint force (Lippitt et al., 1993). Many of the glenoid stabilizing factors have not been individually quantified in humans or other primates, but humans have a shallower glenoid compared with other species, which may reduce their stability ratios at the glenohumeral joint. Though not the only musculoskeletal features contributing to modern human shoulder function, these may be contributing factors in the evolved human propensity for rotator cuff degeneration that could be investigated through a probabilistic computational analysis.

A previous deterministic computational analysis found notable differences between human and chimpanzee rotator cuff muscle forces during horizontal bimanual arm suspension, which highlighted an evolved pathogenesis for human rotator cuff disease in humans (MacLean & Dickerson, 2020). Horizontal bimanual arm suspension is a primarily overhead form of arboreal locomotion in which a subject traverses forward across a horizontally oriented ladder by swinging the arm toward each sequential rung of the ladder in an alternating fashion. During the support phase of this behavior, the arm remains in more than 100° elevation and the elbow extended or mildly flexed (MacLean & Dickerson, 2019). As a probable ancestral locomotor behavior, horizontal bimanual arm suspension and other forms of arborealism are taxing for the modern human shoulder. High‐amplitude muscle forces have been experimentally measured and computationally predicted in humans during the support phase of this behavior (MacLean & Dickerson, 2019; MacLean & Dickerson, 2020). A previous deterministic computational comparison between humans and chimpanzees produced notable differences between species in rotator cuff muscle force predictions for horizontal bimanual arm suspension (MacLean & Dickerson, 2020). Both species had moderate deltoid muscle force predictions. However, chimpanzee rotator cuff muscle forces were moderate and dispersed across the rotator cuff muscle group, while humans were predicted to greatly overload infraspinatus and teres minor with muscle predictions at 100% maximum producing capacity, with limited contribution from supraspinatus and subscapularis. As the human glenohumeral joint is not primarily organized for overhead and climbing behaviors, it is concerning that such high muscle forces were predicted for some rotator cuff muscles. The rotator cuff muscles represent a force couple with the deltoids (Inman et al., 1944). The deltoids and supraspinatus form a superior force vector, while infraspinatus, subscapularis, and teres minor all contribute to an inferior force vector. When balanced, these force couples act to center the humeral head in the glenoid. At the human glenohumeral joint, the rotator cuff muscles are often overpowered by the superior force couple in above shoulder postures, leading to superior migration of the humeral head and a propensity for subacromial impingement and rotator cuff disease (Chopp et al., 2010). Whether this risk for subacromial impingement and rotator cuff is modifiable through moderate changes to the evolutionary musculoskeletal organization is not clear. Perturbation of these force couples, in the form of muscle lines of action, force‐sharing ratios, and glenoid orientation modifications could reduce the overload of human rotator cuff muscles by creating more evenly distributed force sharing across these muscles.

The purpose of this study was to create a probabilistic musculoskeletal model of the chimpanzee and human glenohumeral joints to assess the contribution of different boney features to muscular effort and function of the rotator cuff in the performance of an evolutionarily significant upper extremity task of horizontal bimanual arm suspension. The human deterministic model predicted overloading of the infraspinatus and teres minor muscles, an unsustainable musculoskeletal outcome for climbing in modern humans. The present study aimed to determine if probabilistic modeling of select morphological features of ancestral importance would produce more moderate human muscle forces predictions and greater force sharing between human rotator cuff muscles, resulting in the human muscle force distributions overlapping with the chimpanzee distribution. To this end, functional use of the rotator cuff muscles between species could be seen as merging based on modification of the musculoskeletal features being probabilistically modeled. The morphological features selected for probabilistic analysis were those deemed to have anthropological relevance to the great ape evolutionary divergence and were feasibly and directly scalable within the computational models. These features included rotator cuff origins, deltoid insertion, glenoid inclination, and glenoid stability (Larson, 1995, 1998; Larson & Stern Jr, 1986). It was hypothesized that probabilistic modeling of these musculoskeletal features would change the predicted human and chimpanzee rotator cuff muscle forces during the arm suspension task, such that a notable portion of the human probabilistic distributions would fall within the middle 50% of the chimpanzee distribution. This would modulate the risk for overloading and injury of the rotator cuff in humans. Specifically, it was hypothesized that superior–inferior glenoid inclination alterations and shifts in rotator cuff attachments on the scapula that change muscle lines of action would cause recruitment of all rotator cuff muscles in humans. Higher predictions from subscapularis would likely offload infraspinatus and teres minor muscle predictions, which were recruited to maximum in the deterministic model. Second, it was hypothesized that altering the deltoid insertion on the humerus would modify the superior muscular force couple sharing at the glenohumeral joint by changing the contribution of the supraspinatus muscle. Finally, it was hypothesized that varying the glenoid stability ratios would change recruitment of the rotator cuff muscles by altering the amount of muscular force necessary to compress and center the humeral head into the glenoid.

## 2 MATERIALS AND METHODS

The development of the deterministic human and the chimpanzee glenohumeral models in MATLAB (Mathworks) was previously documented in detail (Dickerson et al., 2007; MacLean & Dickerson, 2020). The chimpanzee model was created with a similar framework as the pre‐existing human (SLAM – Shoulder Loading Analysis Modules, Dickerson et al., 2007) model to facilitate between‐model comparison. The deterministic glenohumeral models are described in detail in a previous publication (MacLean & Dickerson, 2020) and briefly here. The models have three modules—an external dynamic moment module where inverse dynamics are used to generate joint forces and moments, a musculoskeletal geometry module where musculoskeletal geometry is defined, and an internal muscle force prediction module where an optimization routine is used to predict muscle forces. The probabilistic input features modeled in this study exist primarily in the geometry module, except for the glenoid stability ratios, which were one of the constraints of the optimization routine. All muscles are modeled as vector strings from origin to attachment and wrapped around bones using cylindrical and spherical muscle wrapping techniques, to generate more physiologically realistic muscle lines of action. Though modeling muscles as strings oversimplifies muscle physiology and architecture, more detailed models tend to have lower predictive capacity (Buchanan et al., 2004; Scott & Winter, 1991).

Both glenohumeral models are geometrically scalable to emulate known variability in specific musculoskeletal features within each species. This scalability was used to create probabilistic chimpanzee and human models by varying specific glenohumeral morphological traits. The probabilistic models were then used to quantify the effect of the morphological trait variability on rotator cuff muscular activity patterns to quantify the gap between species in rotator cuff function.

### 2.1 Postural, anthropometric, and task‐specific inputs for each model

Both models required postural kinematic data, anthropometric, and task‐specific hand force inputs. As some chimpanzee data are not readily available, some assumptions were made to develop the chimpanzee model. Both models used the same static human right‐arm horizontal arm suspension kinematic inputs, as three‐dimensional chimpanzee kinematic data were unavailable. Modifications were made to the human postural kinematic data to better represent the chimpanzee upper extremity. Anthropometrics were selected from literature sources, to represent an “average” human and chimpanzee. Task‐specific hand forces were conservatively assumed to be the same between species.

The kinematic task input analyzed in both models was a single overhead horizontal bimanual arm suspension cycle. This task represents a likely ancestral behavior for both species. A deterministic static analysis demonstrated that muscle forces produced in the swing phase of the horizontal bimanual arm suspension cycle are minimal, but that differences between species are pronounced in the rotator cuff muscles during support phase (MacLean & Dickerson, 2020). The deterministic predictions of rotator cuff on/off muscle activity were mostly in concordance with electromyographical analyses of chimpanzees, with the exception of subscapularis (no electromyographical activity) and late support infraspinatus and teres minor (notable electromyographical activity) (Larson & Stern Jr, 1986, 2013). As the most pronounced muscles forces occurred during support phase, only this phase was analyzed in the current probabilistic analysis. Three main static instances of the support phase of the arm suspension cycle were used in the analysis: early, mid, and late support. The three static instances of human kinematic data used for the chimpanzee model were inputted into the deterministic chimpanzee model to produce more biofidelic species‐specific joint positions and segment lengths (MacLean & Dickerson, 2020). Chimpanzee model wrist, elbow, and glenohumeral joint centers were iteratively translated to represent appropriate segment lengths of the forearm and arm, using previously reported segment lengths (Schoonaert et al., 2007). Glenohumeral joint position was also translated by shifting the acromion marker to adjust the scapular position to reflect that observed in X‐rays of chimpanzees (Thompson et al., 2020). This process did not alter joint orientation.

Representative average healthy human (mass: 72 kg; height: 1.8 m) and chimpanzee (mass: 45 kg; height: 1.32 m) males were used as the criteria subjects within each glenohumeral model. Subject anthropometry was scaled based on mass and height, according to published data (Schoonaert et al., 2007; Thorpe et al., 1999; Winter, 2009; Zihlman, 1992). The chimpanzee subject represented a theoretical average animal, based on the average data of the subjects used in the publications that provided anthropometric and physiological cross section area data. Hand forces were assumed to be equal to half of body mass in double support phases (early and late support) and total body mass in the single support phase (mid support) of the arm suspension cycle. Based on research on gibbon brachiation, this represented a conservative estimate of substrate reaction forces (Chang et al., 2000).

### 2.2 Geometric scaling and probabilistic variables

#### 2.2.1 Probabilistic input variables

Based on the deterministic results of both the chimpanzee and human glenohumeral models and anthropological literature, specific geometric parameters were chosen as inputs to be modified in the probabilistic analyses. Through previous geometric morphometric analyses, the chosen shoulder features have been associated with arborealism (Larson, 1995, 1998; Larson & Stern Jr, 1986). Features that are presumed to affect glenohumeral arboreal function were selected from primarily the scapula, as well as one from the humerus (Table 1).

**TABLE 1:** Morphological features used as inputs to the probabilistic chimpanzee and human glenohumeral models. The specific computational variable perturbed in each model is also given, along with the anthropological significance for feature selection.

Input feature	Perturbed model inputs	Rationale
Scapular origins of the rotator cuff muscles	Infraspinatus origin [X, Y, Z] Subscapularis origin [X, Y, Z] Supraspinatus origin [X, Y, Z]	Mimic changes to scapular body shape. Associated with rotator cuff lines of action (Larson, 1998)
Deltoid insertion on the Deltoid Tuberosity	Anterior, Middle & Posterior Deltoid insertion [X, Y, Z]	Arm abductors associated with capacity for overhead postures (Larson, 1995)
Glenoid Inclination	Superior–Inferior *Y*‐axis of Glenoid Coordinate System	Superior inclination may optimize joint for overhead behaviors (Larson, 1998)
Intrinsic Stability of the Glenoid	Multiply directional stability ratios by additive factor	Modifies muscle forces required for active joint stabilization (Veeger & van der Helm, 2007)

**FIGURE 1:**
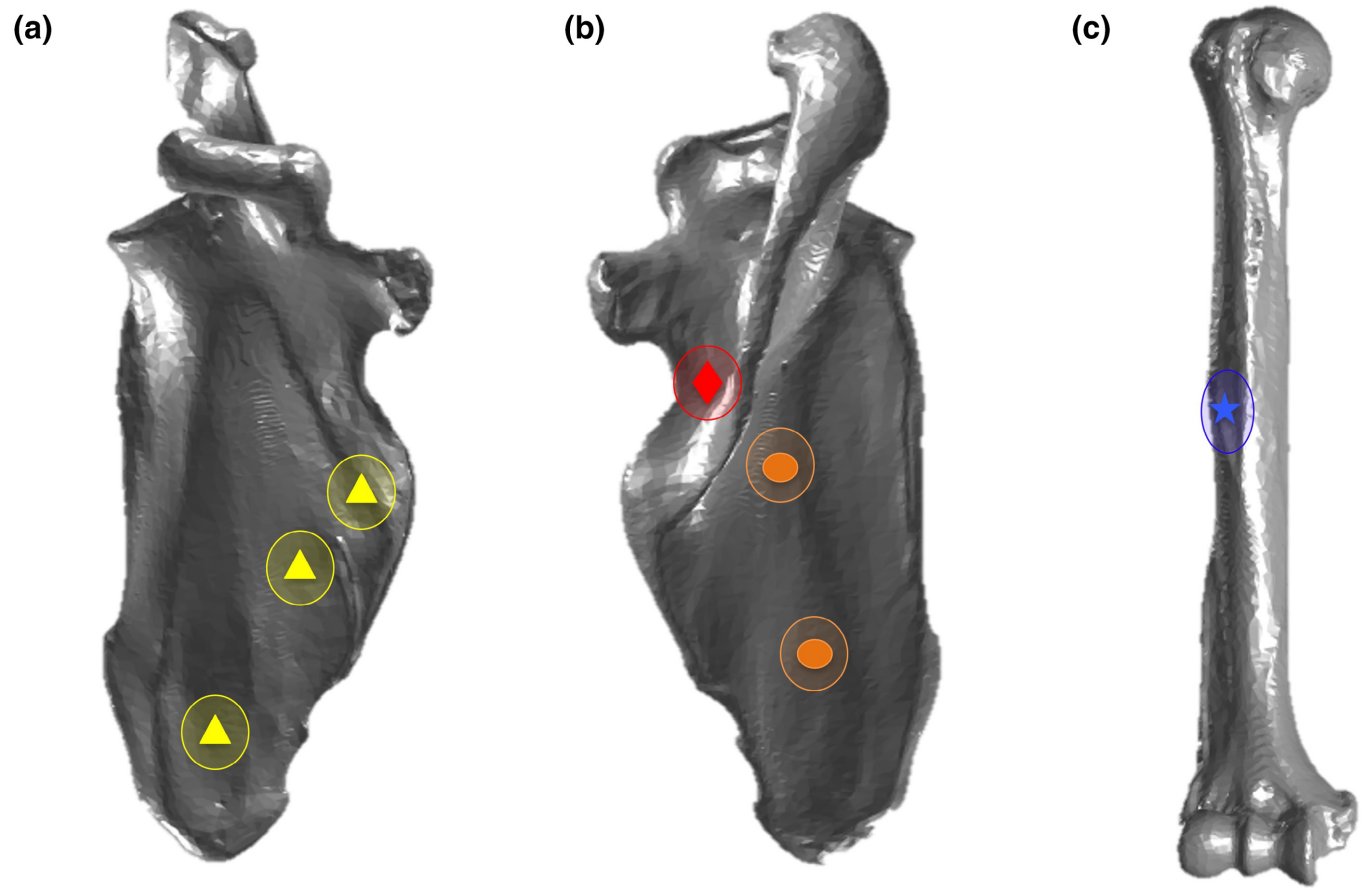
Original three‐dimensional muscle origin or insertion landmark positions on the chimpanzee scapula and humerus are indicated by the symbols on each bone. Included around the symbols is the approximation of the medial–lateral and superior–inferior perturbations that were possible in the probabilistic simulations. Muscle origins and insertions shown represent the (a) subscapularis (triangles) origin, (b) infraspinatus (circles) and supraspinatus (diamond) origins and (c) deltoid (star) insertion that were varied in the probabilistic models.

The three‐dimensional coordinates of infraspinatus, supraspinatus, and subscapularis muscle origins and deltoid muscle insertion relative to segmental rotation points were explicitly coded into both species models and were used to perturb each of these muscle positions (Figure 1). All landmarks are three‐dimensional (X, Y, Z), representing anterior–posterior, medial–lateral, and superior–inferior position of each muscle origin or insertion. These origins and insertions are locally expressed as a percentage of longitudinal bone length, in the coordinate system of the specific bone (Hogfers et al., 1987; Makhsous, 1999). Each muscle origin or insertion was varied along three dimensions—superior–inferior, medial–lateral, and anterior–posterior.

The infraspinatus muscle and subscapularis muscle were modeled with multiple elements in both the chimpanzee and human model, to reflect known muscular geometry. These elements represented portions of each muscle with distinct biomechanical paths (Hogfors et al., 1987). The infraspinatus muscle was modeled with an upper and lower element. The subscapularis muscle was modeled with upper, middle, and lower elements. To maintain probabilistic simulation parsimony, only the upper infraspinatus and upper subscapularis were directly probabilistically modeled. Each of the original three‐dimensional coordinates of the other infraspinatus and subscapularis elements were converted into percentage differences from the upper element's three‐dimensional coordinates. This formulation required these muscles to act as a unit in the probabilistic model.

**FIGURE 2:**
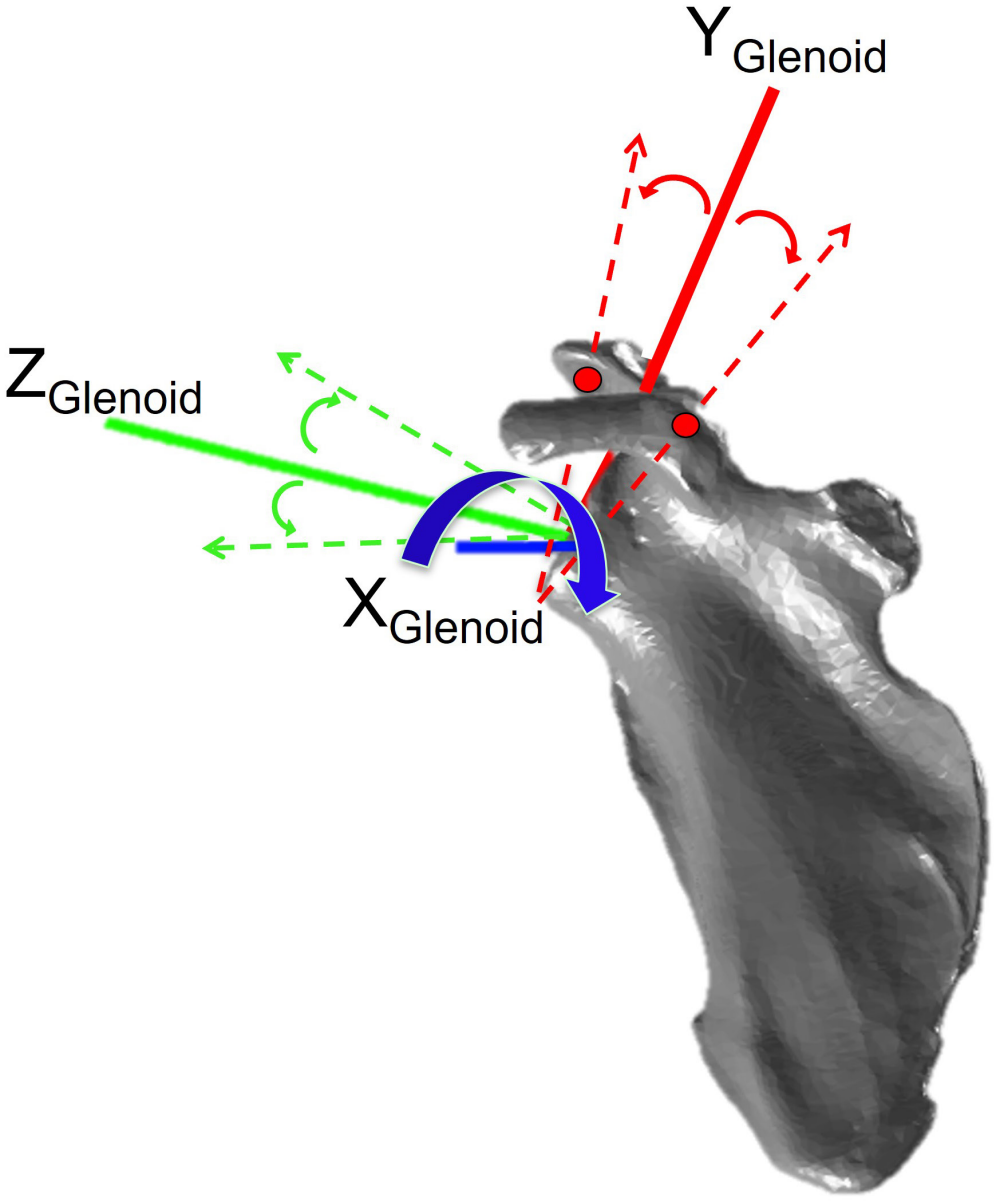
Glenoid inclination was varied by rotating the angle of the glenoid. For the probabilistic input of glenoid inclination, the superior point (red circle) of the y‐axis of the glenoid coordinate system was modified to shift the representation of the inclination plane of the glenoid.

Glenoid inclination was varied by altering the angle of the superior–inferior *y*‐axis vector of the local glenoid coordinate system (Figure 2). This vector of the glenoid coordinate system comprises the superior landmark of the glenoid (vector tip) and centroid of the glenoid (vector tail). To change the angle of this vector, the superior landmark of the vector—which identified the superior point of the glenoid in global three‐dimensional (X, Y, Z) coordinates—was shifted through a rotation about the anterior–posterior *x*‐axis (Figure 2).

**FIGURE 3:**
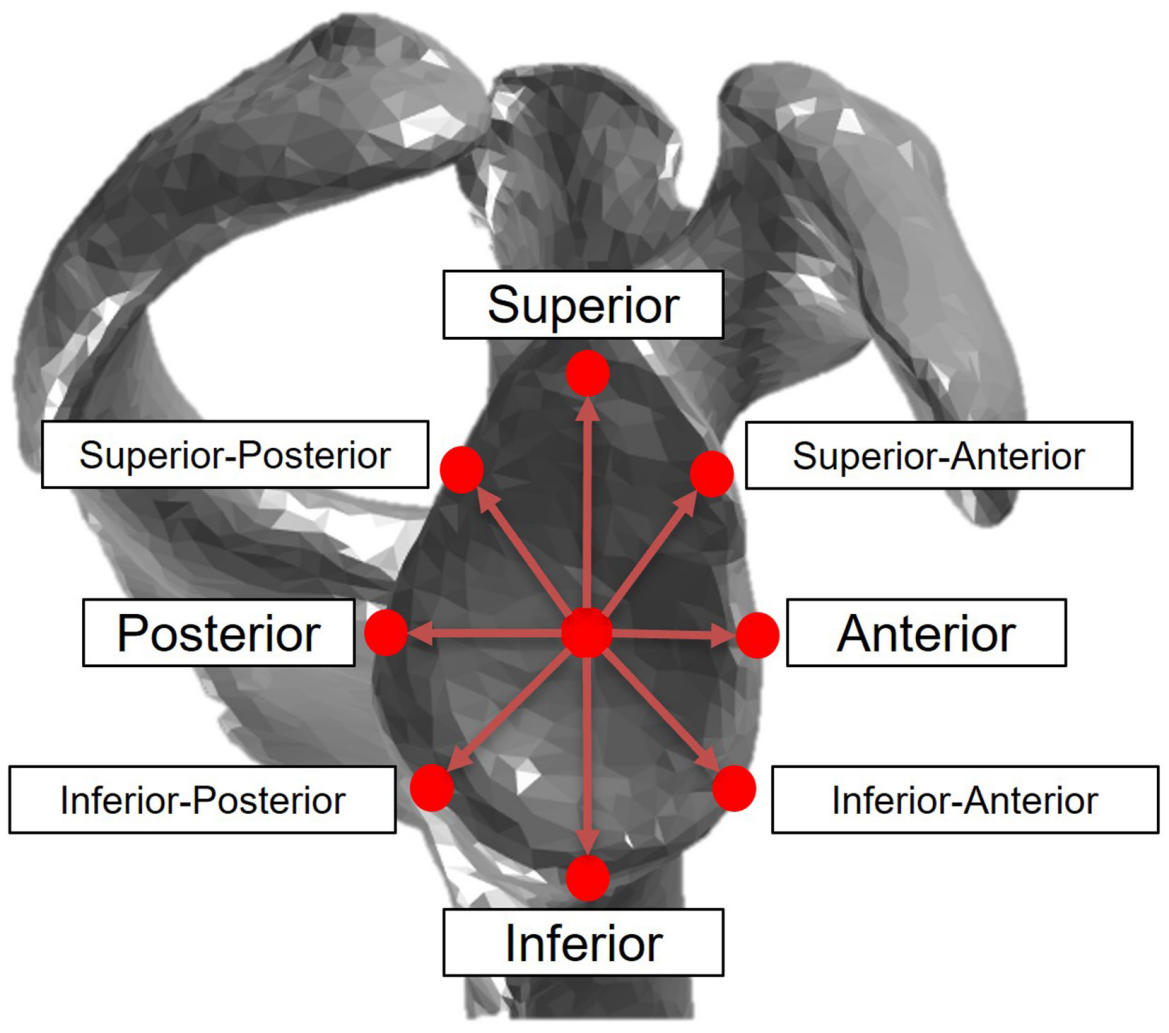
Each of the eight mathematically modeled stability ratio directions is shown around the rim of a chimpanzee glenoid. Stability of both the human and chimpanzee glenohumeral joint can be mathematically represented as eight direction‐specific ratios of dislocating shear forces to stabilizing compressive forces. Probabilistic distributions were used to perturb stability in all eight‐directions.

All eight directional glenoid stability ratios in both the chimpanzee and human model were altered to have an additive factor. Stability of the glenohumeral joint is complex and dependent on many physiological and biomechanical factors (Veeger & van der Helm, 2007). In its simplest form, stability of the glenohumeral joint is dependent on the total dislocating shear force being less than the total compressing force at the joint. As the glenohumeral joint is anisotropic, the tolerance for dislocating shear forces is different depending on the direction of the force. To simplify the computational modeling of glenohumeral stability, it can be mathematically modeled as a series of eight multidirectional ratios of shear dislocating forces that can be applied in the glenoid for a given compressive stabilizing force (Figure 3) (Lippitt et al., 1993). As humans and chimpanzees have similarly shaped glenoids (Macias & Churchill, 2015), differences in directional glenoid stability between species are assumed to be due to glenoid depth only. Based on medical image comparisons of the chimpanzee and human glenoid, the chimpanzee glenoid is approximately 13.13% deeper than the human glenoid (MacLean, 2018). Therefore, an additive factor of 13.13% was explicitly coded into the deterministic chimpanzee glenohumeral model stability ratios, in all eight directions. This value represented a 13.13% greater capacity to withstand a shear force for a given compressive force in the chimpanzee glenoid, compared with the deterministic human glenohumeral joint model. In the probabilistic model, baseline stability values in each model were assumed to represent mean baseline values, with distributions applied to vary intrinsic stability of the glenoid by a percentage of baseline stability. Human stability ratios were varied around the original baseline values, and chimpanzee stability ratios were varied around a 13.13% change above the human baseline values. All eight directional stability ratios were probabilistically modeled together as a unit, in both the human and chimpanzee models.

#### 2.2.2 Probabilistic input distributions

Distribution ranges for each input variable were determined through manipulation of the deterministic models for each species. The manipulations of the deterministic model were used to establish an appropriate standard deviation for each muscle attachment input in the probabilistic software. This process ensured that each musculoskeletal variable stayed within reasonable anatomical boundaries of the bones and joints during the probabilistic analysis, where most iterations in the simulation would fall within 3 standard deviations. For muscle insertions and origins, boundaries were estimated on each bone through an iterative process within both the human and chimpanzee model. Values employed in previous, analogous work on humans and the anatomical three‐dimensional size of each bone were used to develop standard deviation boundaries that kept muscle origin and insertions within the anatomical bone surface in all three directions (Chopp‐Hurley, 2015). Therefore, the standard deviations employed in the origin and insertion of rotator cuff and deltoid muscle perturbations were representative of ranges documented in humans. Three‐dimensional X, Y, and Z coordinates of the rotator cuff origins and deltoid insertion were shifted to determine anatomically realistic variation and standard deviations on the bone surface along superior–inferior, medial–lateral, and anterior–posterior directions (Table 2).

**TABLE 2:** Means, standard deviations (SD) for all the input variables used in the probabilistic simulations. Muscle insertions and origins means are presented as a percentage of bone length. Muscle origins and insertions on both the scapula or humerus were modified three‐dimensionally in the superior–inferior, medial–lateral, and anterior–posterior directions.

Input feature	Human mean (SD)	Chimpanzee mean (SD)
Infraspinatus origin	Anterior–Posterior: −0.106 (0.010)	Anterior–Posterior: −0.200 (0.010)
	Medial–Lateral: 0.240 (0.035)	Medial–Lateral: 0.300 (0.035)
	Superior–Inferior: 0.607 (0.035)	Superior–Inferior: 0.587 (0.035)
Subscapularis origin	Anterior–Posterior: −0.030 (0.010)	Anterior–Posterior: −0.0615 (0.010)
	Medial–Lateral: 0.307 (0.035)	Medial–Lateral: 0.162 (0.035)
	Superior–Inferior: 0.523 (0.035)	Superior–Inferior: 0.643 (0.035)
Supraspinatus origin	Anterior–Posterior: −0.050 (0.010)	Anterior–Posterior: −0.051 (0.010)
	Medial–Lateral: 0.315 (0.035)	Medial–Lateral: 0.125 (0.035)
	Superior–Inferior: 0.360 (0.035)	Superior–Inferior: 0.460 (0.035)
Deltoid insertion	Anterior–Posterior: −0.004 (0.035)	Anterior–Posterior: 0.0315 (0.035)
	Medial–Lateral: 0.064 (0.010)	Medial–Lateral: 0.0415 (0.010)
	Superior–Inferior: 0.369 (0.035)	Superior–Inferior: 0.346 (0.035)
Glenoid inclination (cm)	115.12 (0.3)	104.78 (0.3)
Stability ratio (%)	0.0 (4.0)	13.13 (4.0)

The range of variation in glenoid inclination, in degrees, was informed by determining the angular change of inclination associated with each iterative shift of the superior point of the glenoid. Based on this relationship, a standard deviation was determined for the glenoid superior point from previous research on variation of glenoid inclination in human and chimpanzee populations (Chopp‐Hurley, 2015; Larson, 1995, 2007). The standard deviation applied to the global mediolateral position of the superior glenoid landmark, in centimeters, created an approximately total 20° angular range of glenoid inclination within three standard deviations of the probabilistic distribution for both models (Table 2) (Larson, 2007).

For stability ratios, no data exist on chimpanzee variability. The percentage difference in stability ratios between the two species was used to guide an appropriate variation for both species. The standard deviation applied to each species represented a total variance of 12% in stability in all eight directions. The variance value was a conservative estimate that prevented the stability ratio distribution from exceeding the difference between species (Table 2).

Standard deviations were kept the same between the two models for all probabilistic input variables. This was done to reduce the possibility of different distributions between the human and chimpanzee model introducing a confounding factor. Maintaining the same variability in both models prevented any probabilistically modeled input from having an artificially greater effect in one model over the other.

#### 2.2.3 Probabilistic output variables: Rotator cuff muscle forces

To focus the probabilistic analysis, outputs analyzed from the models were reduced from all possible deterministic model outputs. To emphasize potential differences between species in rotator cuff function, the probabilistic analysis focused on three static instances of the support phase of the arm suspension cycle (early, mid, and late support), where rotator cuff muscle forces were relatively higher in both species, and thus interspecies differences would be most pronounced. A total of seven outputs were examined in the three static instances of right‐arm horizontal bimanual arm suspension support phase, representing the entirety of the rotator cuff. Cumulative distribution functions were obtained for the lower and upper infraspinatus muscle elements, lower, middle, and upper subscapularis muscle elements, supraspinatus muscle, and teres minor muscle.

### 2.3 Probabilistic musculoskeletal modeling simulations

The probabilistic models entailed interfacing NESSUS Version 7.01 (Southwest Research Institute) probabilistic analysis software with the MATLAB® (Mathworks) software environment in which the deterministic human and chimpanzee glenohumeral models exist. NESSUS software allows distributions of multiple inputs and model parameters to be applied at once as covarying random variables. Distributions of each morphological feature were applied by mapping in NESSUS to the parameters defined within the deterministic model (Chopp‐Hurley, 2015). This process created 99% distributions for the desired output variables of rotator cuff muscle forces. Monte Carlo simulations were chosen as they are most common and considered a gold standard technique (Langenderfer et al., 2008).

To create a probabilistic model, each of the input variables was set to the mean value (Table 2) in the original chimpanzee and human deterministic models. Means and standard deviations of each of the input variables (Table 2) were set in NESSUS for both the human and chimpanzee models and used to form a normal (Gaussian) distribution. Normal distributions were selected, as they are commonly used in science and engineering, and typically best represent the true distribution of biological phenomena (Choi et al., 2007). Each variable was then mapped to the line of code in the MATLAB glenohumeral model program representing the mean value. All input variables (each muscle attachment site, glenoid inclination, glenoid stability ratio) were randomly perturbed within their normal distribution simultaneously in NESSUS. By assessing all morphological variables concurrently, the simultaneous effect of all features' variability on rotator cuff muscle forces was determined, allowing for intentional interaction between input variables.

#### 2.3.1 Sensitivity factors

Of importance in the analysis was the effect of each of the input variables on the output muscle forces, which was evaluated in nondimensionalized space. Monte Carlo probabilistic simulations produce absolute sensitivity factors that indicate how the input variables contribute to the distribution of the output variables, normalized to a discrete probability level (Laz & Browne, 2010). Absolute sensitivity factors indicate how much the mean of each input variable affected the output distribution (Equation 1). These values are often averaged across entire movement cycles and were therefore averaged across the entire support phase of the arm suspension cycle (Laz & Browne, 2010).

Equation 1: Calculation of absolute sensitivity factors for an output distribution from Monte Carlo simulations as determined by perturbation of the mean. μ and *σ* are the mean and standard deviation for a given input variable, and p is the specified probability level.

$$S_{\mu }=\frac{\partial p\;\sigma_{i}}{\partial \mu_{i}\;p}$$

#### 2.3.2 Probabilistic simulations

A series of Monte Carlo analyses were run at 2500 iterations for each of the output normalized muscle forces (two infraspinatus, three subscapularis, one supraspinatus, and one teres minor), in the three static support instances of early, mid, and late support, for both the human and chimpanzee glenohumeral models. A 2500 iteration Monte Carlo sufficiently coincided on the correct solution in earlier shoulder simulations (Chopp‐Hurley, 2015; Langenderfer et al., 2008). A total of 42 Monte Carlo analyses were completed, 21 for each species model. Each Monte Carlo analysis created a cumulative distribution function, with probabilities at 11 specified levels—0.01, 0.1, 0.2, 0.3, 0.4, 0.5, 0.6, 0.7, 0.8, 0.9, and 0.99—and sensitivity factors for each of the input parameters at each probability level.

#### 2.3.3 Covariance

Musculoskeletal features may covary. A change in one feature may have an associated change in a related musculoskeletal feature. The chosen probabilistic approach allowed the full range of each input distribution to be considered in combination with the full range of all other input feature distributions. This approach meant that combinations of features may be considered that are not necessarily biologically realistic. However, the influence of covariance for several morphological shoulder features on subacromial geometry was previously determined to be very low (Chopp‐Hurley, 2015). Thus, covariance was assumed to be negligible in the present study and not incorporated into the model.

### 2.4 Data analysis

Human and chimpanzee muscle forces of the rotator cuff (supraspinatus, infraspinatus, subscapularis, teres minor) from the three static (early, mid, and late) support instances were analyzed as outputs of the probabilistic Monte Carlo analysis in NESSUS. Muscle forces were reported as a percentage of maximum muscle force, dependent on known muscle physiological cross‐sectional area (Dickerson et al., 2007; MacLean & Dickerson, 2020). The 1st (minimum), 25th, 50th (median), 75%, and 99th (maximum) probability levels were extracted from resultant cumulative distribution functions to compare predicted 99% confidence interval ranges for the input variables that were probabilistically modeled of muscle forces between chimpanzees and humans. Overlap in confidence intervals of chimpanzees and humans was used to indicate quantitative mergence in rotator cuff muscle forces between species. Specifically, overlap of muscle force distributions was examined to determine if the inner 50th percentile or outer 25th percentiles of human distributions overlapped with the chimpanzee distributions and the median chimpanzee muscle forces. Particular attention was given to whether predictions reduced the overloading of human rotator cuff muscles and drew the human predictions closer to chimpanzee predictions for rotator cuff muscle forces.

To test the relationship between each morphological feature and the outputs, sensitivity factors were extrapolated from each of the Monte Carlo analyses. Sensitivity factors were produced for each of the input variables, at each discrete probability level, for each Monte Carlo analysis on each of the seven output variables, for all three static support instances. Sensitivity factors were averaged across the probability levels and then across each of the instances of the climb cycle support phase (Laz & Browne, 2010). The final sensitivity factors represent the averaged effect of each of the 14 input variables on each of the seven output variables.

## 3 RESULTS

**FIGURE 4:**
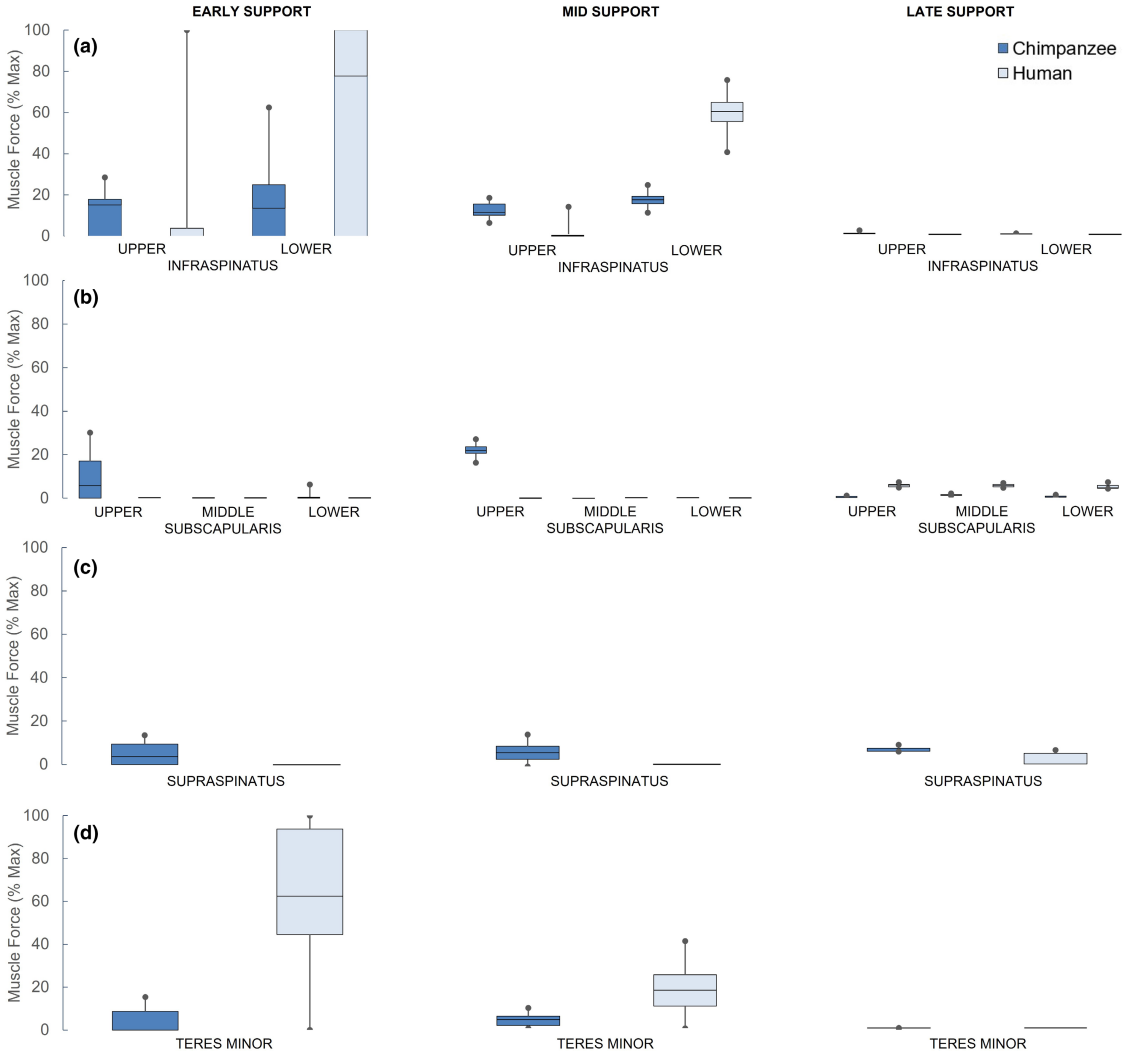
Cumulative distribution results of the Monte Carlo analysis for all four rotator cuff muscle force predictions. Cumulative distributions are presented for (a) Upper and lower infraspinatus; (b) upper, middle, and lower subscapularis; (c) supraspinatus; (d) teres minor. Each cumulative distribution represents the predicted muscle activity of an individual muscle as a percentage of its maximal force‐producing capacity. Results are shown for the three static instances of the support phase of the climb cycle. The box‐and‐whisker plots represent a 99% confidence interval for each muscle force. The inner two quartiles within the box represent the 25%–75% percentile of the distribution, with the line in the box representing the 50th percentile, and the whiskers representing the outer quartiles.

Cumulative distribution functions were used to determine overlap between species in rotator cuff muscle force distributions. Muscle forces are presented as a percentage of estimated maximum muscle force (Figure 4).

### 3.1 Muscle force distributions

The confidence intervals of late support rotator cuff muscle activity were low for both species. All confidence intervals fell within a narrow range of 0%–8% maximal force. Consequently, the late support confidence intervals for all rotator cuff muscles for both the chimpanzee and human models overlapped (Figure 4).

Muscle activity was low to moderate in mid support, except for the human lower infraspinatus muscle. Subscapularis and supraspinatus muscle forces were only predicted to be above 0% in the chimpanzee distribution. Chimpanzees had a teres minor distribution with median 4.1% (range: 0%–9.62%), whereas humans had a distribution with median 18.06% (range: 0%–41.24%), with 75% of this distribution falling above the chimpanzee distribution. While chimpanzee infraspinatus muscle force distributions were low to moderate, the human lower infraspinatus distribution was high, with a median value of 60.23% (range: 40.26%–75.69%). This was much greater than the chimpanzee distribution and indicative of overloading infraspinatus.

Most muscle activity was in early support, where differences in notable rotator cuff muscle forces existed between species in the probabilistic simulation. Prediction ranges for the human rotator cuff muscles of infraspinatus and teres minor were much greater than the chimpanzee, with predictions including 100% muscle force in early and mid support (Figure 4a,d). The confidence intervals for the human upper and lower infraspinatus and teres minor in early support had median values of 0% (range: 0%–100%), 77.71% (range: 0%–100%), and 62.48% (range: 0%–100%), respectively. Due to these large confidence intervals, they overlapped with the predicted range of forces for respective chimpanzee muscles, despite the chimpanzee confidence intervals being much lower (Figure 4a,d). However, the lower 50% of the human upper infraspinatus distribution was predicted to be 0%. Combined with the entire range of this muscle element, this did not appear to functionally overlap with the moderate chimpanzee distribution of upper infraspinatus muscle forces. Similarly, 50% of the human lower infraspinatus distribution was predicted to be either 0% or 100%, and the median value was above the entire distribution of the chimpanzee. Both human infraspinatus element distributions indicate a large amount of maximal loading and unloading of this muscle that does not functionally overlap with chimpanzee predictions. The lower end of the human teres minor muscle distribution overlapped with the chimpanzee distribution, but more than 50% of the human teres minor distribution was above the entire chimpanzee distribution, which does not demonstrate a functional overlap or notable reduction of human muscle force predictions.

Only chimpanzees were predicted to activate the subscapularis and supraspinatus muscles in most of support phase. Activation of subscapularis was mostly restricted to the upper element in chimpanzees in early, 5.72% (range: 0%–57.71%), and mid support, 23.51% (range: 17.82%–28.51%). Human subscapularis activity was 0%, with the exception of late support (Figure 4b). A small bout of subscapularis activity was predicted in the lower element, though the median was 0% (range: 0%–6.31%). Similarly, the only human supraspinatus activity confidence interval that was non‐zero was in late support and was very low, with a mean of 0% (range: 0%–6.24%). Chimpanzees were predicted to have consistent, but low, activity from supraspinatus throughout the support phase (Figure 4c).

### 3.2 Sensitivity factors

**FIGURE 5:**
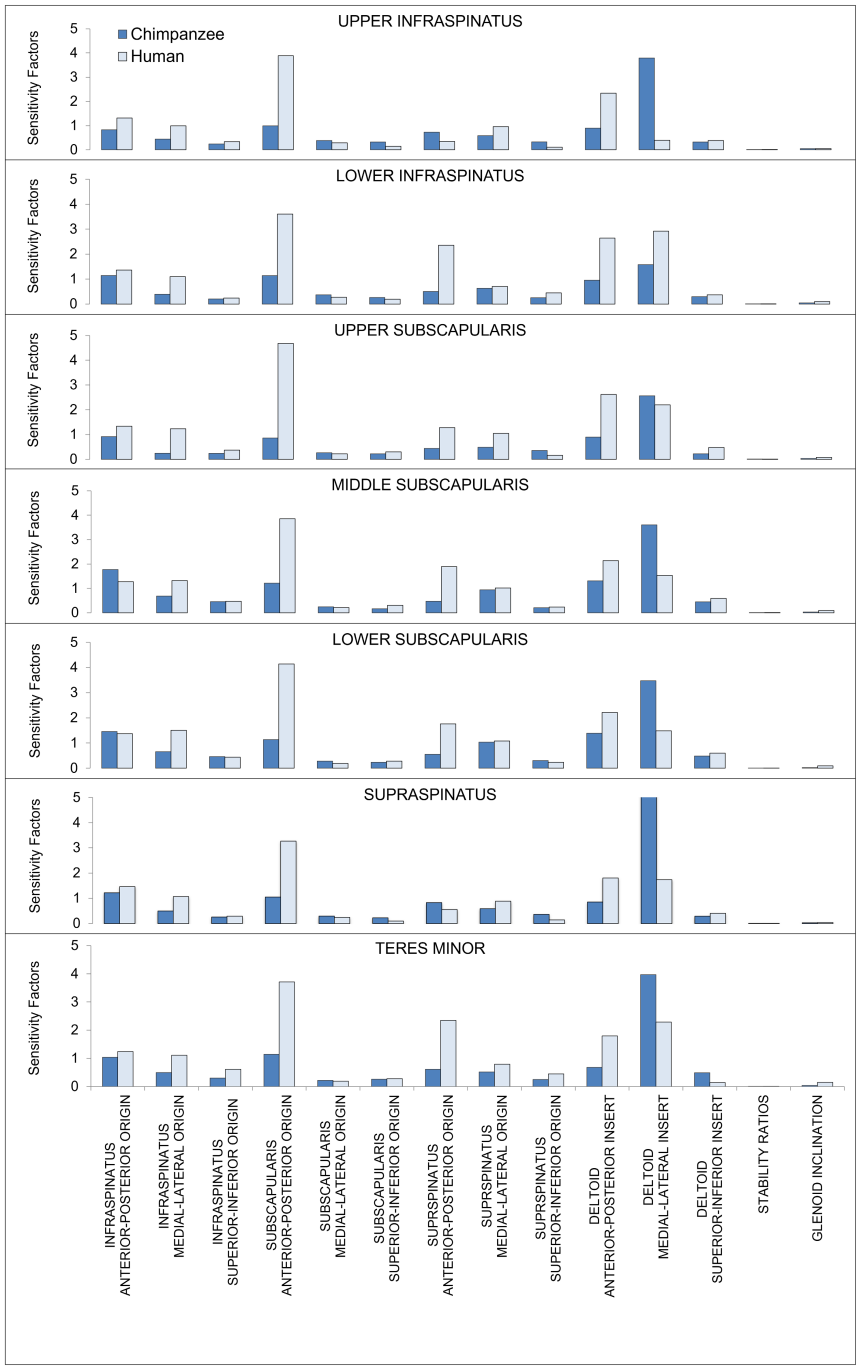
Sensitivity factors of each of the seven output muscle elements to distributions of the input variables produced by the Monte Carlo analysis. The magnitude of the sensitivity factors indicates how strongly that input variable contributed to the rotator cuff output distribution. The input variables are the three‐dimensional distributions of the infraspinatus origin, subscapularis origin, the supraspinatus origin, and deltoid insertion, and glenohumeral stability ratios and the glenoid inclination.

Magnitude of sensitivity factors varied greatly across all inputs, indicating which variables had a stronger effect on rotator cuff muscle forces. Averaged sensitivity factors for each of the predicted rotator cuff muscle forces are presented in Figure 5. Greater values indicate that a specific input more influentially perturbed the mean of the resultant output variable distribution.

The geometric inputs with the greatest effect differed between the two species models. The anterior–posterior perturbation of the subscapularis origin consistently had the greatest influence on human model outputs. In the chimpanzee model, the deltoid insertion medial–lateral *z*‐component perturbations most influenced all the rotator cuff outputs (Figure 5). Changes to glenoid inclination and stability ratios had negligible effects on all output distributions (Figure 5). Shifts in muscle insertions and origins along the superior–inferior length of both the scapula and humerus had a smaller effect than the anterior–posterior and medial–lateral perturbations in both models.

## 4 DISCUSSION

The presented probabilistic modeling of the chimpanzee and human glenohumeral joint permits population‐level comparisons between the two closely related species. The study hypothesis was mostly not supported. Muscles that were predicted to not contribute to the suspension task deterministically were also predicted to not contribute stochastically. The probabilistic simulations limited observed human model rotator cuff muscle distributions to overloading infraspinatus and teres minor. Conversely, probabilistic simulations predicted low‐to‐moderate rotator cuff distributions in the chimpanzee model. While large distributions were produced for the human infraspinatus and teres minor, causing overlap between chimpanzees and humans in these two muscle forces, no functional species overlap existed in muscle force distributions, as a large percentage of the human distributions remained near or at either 0% or 100% maximum force. Perturbations of rotator cuff muscle origins on the scapula contributed to resultant muscle force distributions, while glenoid inclination and stability ratios negligibly influenced predicted rotator cuff muscle forces. Though this research did not demonstrate functional overlap between the two closely related species, it identified morphological features that affect function, substantiates previous evolutionary morphometric comparisons, and implicates evolutionary adaptations in the development of human glenohumeral musculoskeletal disorders.

### 4.1 Predicted rotator cuff distributions

Human rotator cuff muscle forces remained different than chimpanzees, and higher, denoting the difficulty of the horizontal bimanual suspension task in modern humans. The human infraspinatus and teres minor activated as high as 100% of muscle force within the 99% confidence interval. Corresponding chimpanzee confidence intervals were typically below 40% maximal force. Though possibly ancestral, weight‐bearing horizontal bimanual arm‐suspension places the modern human glenohumeral joint under immense duress, requiring high muscle forces as a percentage of maximal force producing capabilities to counter external forces (Lewis et al., 2001; MacLean & Dickerson, 2019). Instead of moving toward more chimpanzee‐like muscle forces, the human stochastic model trended toward overloading the infraspinatus and teres minor. This suggests none of present probabilistic modeling choices modified the human shoulder toward ancestral abilities or lessened the burden of suspended locomotion on the modern human shoulder.

The width of the human infraspinatus and teres minor confidence intervals indicates an interplay between minimizing and overloading each of the muscles. This may denote the importance of these muscles to climbing and weight‐bearing activities and an overlap in function. From electromyographical studies, infraspinatus and teres minor are active in chimpanzees during the support phase of arm suspension, (Larson & Stern Jr, 2013; Larson & Stern Jr, 1986). High infraspinatus electromyographical activity has also been observed in humans during support phase, though not maximally (MacLean & Dickerson, 2019). The human rotator cuff, while overall very similar to the chimpanzee, is more infraspinatus‐dominant (Mathewson et al., 2014; Sonnabend & Young, 2009; Thorpe et al., 1999). While teres minor constitutes the smallest rotator cuff muscle, it has a greater PCSA in humans than other primates (Carlson, 2006; Mathewson et al., 2014; Thorpe et al., 1999). Many muscles at the human shoulder have low force producing capabilities and lines of action that are not optimized for overhead tasks (Carlson, 2006; Larson & Stern Jr, 2013; Lewis et al., 2001). The increase in size as a proportion of the total rotator cuff of both infraspinatus and teres minor in humans may explain their greater dominance in the computational human model, as the cost is lower in an optimization routine with a stress‐based objective function. However, the apparent inability to shift loads to alternate glenohumeral muscles, including those beyond the rotator cuff muscles, in the modern human shoulder indicates reduced mechanisms to prevent rotator cuff muscle overload in high force, overhead behaviors. It is also notable that maximal infraspinatus and teres minor muscle forces were predicted in the human model, despite conservative hand forces. Previous research on gibbons demonstrated average substrate reaction forces of 1.5 times body mass during brachiation (Chang et al., 2000). The current study has likely underestimated the magnitude of force distributions experienced at the joints of the upper extremity and thus, the muscle force predictions.

Subscapularis and supraspinatus were rarely predicted to be active in the human stochastic model, with no perturbations increasing the probability of activation of either muscle. Though the available experimental data are limited, subscapularis is not considered a notable active contributor in many arm‐swinging and suspension tasks in non‐human primates, beyond electromyographical activity in swing phase (Larson & Stern Jr, 1986, 2013). Though no experimental electromyographical human subscapularis data exist, that this muscle is not very active in other primates may explain why the present probabilistic perturbations did not result in the recruitment of subscapularis in the human model to offload infraspinatus. However, supraspinatus has an important role in climbing as a joint stabilizer and arm elevator (Larson & Stern Jr, 1989; Larson & Stern Jr., 1992) and is active in humans and at low levels in chimpanzees during the climbing support phase (Larson & Stern Jr, 1986, 2013; MacLean & Dickerson, 2019). The lack of supraspinatus activity in the human model, despite the probabilistic muscle attachment perturbations of the superior force couple of the supraspinatus and deltoid, may signify the decreasing evolutionary role of supraspinatus at the glenohumeral joint, while also reflecting a small moment arm. The human supraspinatus has a small mass and PCSA (Table 3) (Mathewson et al., 2014), possibly to accommodate the narrower human subacromial space through which the supraspinatus tendon passes (Voisin et al., 2014). Compared with other primates, the human deltoids have a much greater volume and PCSA proportionally to the rotator cuff (Carlson, 2006; Thorpe et al., 1999). This difference reduces the role for the supraspinatus in arm elevation tasks, such as arm suspension (Santago et al., 2015). Supraspinatus is also a glenohumeral joint stabilizer (Larson & Stern Jr., 1992). However, to counter the superior, elevating action of the deltoids, the computational model optimization routine would select a mathematically larger and stronger muscle that could geometrically perform a similar stabilizing role, such as infraspinatus (Table 3).

**TABLE 3:** Muscle PCSA for the deltoid and rotator cuff muscle elements included in the force prediction module of the human and chimpanzee deterministic and probabilistic glenohumeral models. Absolute values are given, as well as relative to the total body mass of the human and chimpanzee individuals used in each model.

Muscle	Absolute PCSA (cm^2^)		Relative PCSA to body mass (cm^2^/kg)	
	Human^a^	Chimpanzee^a^	Human^a^	Chimpanzee^a^
Deltoid middle	7.42	28.95	0.103	0.643
Deltoid posterior	4.29	11.06	0.060	0.246
Deltoid anterior	8.84	12.10	0.123	0.269
Infraspinatus 1 (upper)	6.37	11.08	0.088	0.246
Infraspinatus 2 (lower)	7.67	13.34	0.107	0.296
Subscapularis 1 (upper)	2.83	11.19	0.039	0.249
Subscapularis 2 (middle)	3.72	14.71	0.052	0.327
Subscapularis 3 (lower)	5.10	20.17	0.071	0.448
Supraspinatus	3.15	19.92	0.044	0.443
Teres minor	2.81	5.48	0.039	0.122

^a^ Human PCSA was acquired from Makhsous, 1999. The human data were measured from elderly individuals. As PCSA decreases with age, the values presented here have been doubled to more accurately represent the PCSA of younger, healthy human adults (Dickerson et al., 2007). Chimpanzee PCSA was acquired or derived from Carlson, 2006; Kikuchi, 2010; Michilsens et al., 2009; Oishi et al., 2009; Thorpe et al., 1999. The listed PCSA values for both species are averages of the PCSA values cited in the listed literature sources. Chimpanzee subscapularis and infraspinatus were provided as whole muscle PCSA, not partitioned. Therefore, the total PCSA for each of these muscles was partitioned into their elements based on known human percentages of total infraspinatus and subscapularis. PCSA data relative to body mass were used in both models.

### 4.2 Rotator cuff sensitivity to input distributions

Perturbations to medial–lateral and anterior–posterior muscle insertions and origins had the most influence on resultant rotator cuff muscle forces. Muscle origins and insertions affect the orientation of muscle fibers as they cross the joint (Larson & Stern Jr, 2013). Medial–lateral and anterior–posterior attachments may also alter the shearing forces produced by muscles around the glenoid, resulting in a redistribution of the muscle forces necessary to stabilize the glenohumeral joint (Inman et al., 1944). While muscle attachment positions were strong modifiers of muscle force outputs (Biewener, 1990; Nussbaum & Chaffin, 1996), they did not cause distributions that indicated that humans could merge toward chimpanzee glenohumeral biomechanics. Rather, the probabilistically modeled muscle origins and insertions reinforced existing biomechanical patterns in each species. Humans maintained selection bias for infraspinatus and teres minor only. These results highlight the complexity of the form and function relationship and challenge the inference that modest changes to isolated musculoskeletal features strongly associate with evolutionary species‐specific function and locomotion (Carlson, 2005; Green et al., 2015; Larson, 1998; Larson & Stern Jr, 2013; Young, 2006). As isolated features, rotator cuff muscle origin and insertion along the medial–lateral width and anterior–posterior depth of the scapula do modestly affect within‐species muscle force‐sharing strategies, but do not strongly affect functional behavioral outcomes such as brachiating capacity. Greater adaptations to the glenohumeral system may be required to notably modify functional outcomes through musculoskeletal form.

The human and chimpanzee models had different sensitivities to the probabilistic inputs. Despite being a fairly inactive muscle, the human model was most sensitive to anterior–posterior perturbations of the subscapularis origin, while the chimpanzee model was most sensitive to the medial–lateral perturbations of the deltoid insertion. The two models were designed to be analogous, except for the musculoskeletal geometry component of the model where bone and muscle geometry diverge (MacLean & Dickerson, 2020). The difference in sensitivity is likely a response to species differences in force sharing due to muscle biomechanics and physiological properties across the glenohumeral joint. Chimpanzees have a larger rotator cuff PCSA and size relative to the deltoid muscles compared with humans (Table 3) (Carlson, 2006; Thorpe et al., 1999). In turn, greater force sharing and exchange are possible between the rotator cuff and deltoids (Santago et al., 2015). For this reason, modifying the deltoid insertion in the medial–lateral direction likely alters the contribution of the rotator cuff to the chimpanzee glenohumeral joint muscle moment. The sensitivity of all human outputs to subscapularis origin medial–lateral position is less clear. Subscapularis has been previously shown to contribute to numerous rotator cuff muscle force distributions in probabilistic modeling of the human model (Chopp‐Hurley et al., 2014). Therefore, the geometry of subscapularis may influence the compensations of other rotator cuff muscles, such as loading and offloading of infraspinatus and teres minor, to glenohumeral stability. This may be more pronounced in humans due to the reduced force producing capacity of the rotator cuff muscles (Mathewson et al., 2014). Previous computational musculoskeletal models have demonstrated limited functional sensitivity to muscle origin sites when compared with muscle insertion sites (O'Neill et al., 2013; van Beesel et al., 2021, 2022). Therefore, that a muscle origin had the greatest effect on the human model probabilistic distributions is even more surprising and may be indicative of specific morphological and muscular constraints of the human shoulder.

### 4.3 Implications for human evolution and modern human glenohumeral function

The action of the rotator cuff and deltoid muscles is similar between chimpanzees and humans, with some key evolutionary adaptations that modify force sharing and thus pathomechanical pathways about the glenohumeral joint. The rotator cuff and deltoids have been emphasized as force couples that provide synergistic stabilization to the glenohumeral joint for a versatile set of behaviors (Inman et al., 1944). However, in chimpanzees and other primates, this role may be secondary to active weight‐bearing locomotor roles that distinguish these species from the musculoskeletal form of the human shoulder (Larson, 1998; Larson & Stern Jr, 1986; Roberts, 1974). Owing to non‐weight‐bearing usage, the human rotator cuff is much smaller in size and absolute PCSA compared with the chimpanzee (Carlson, 2006). Yet, the human deltoids have remained large proportionally to the rotator cuff (Table 3) (Carlson, 2006; Santago et al., 2015). Changes to the orientation of the glenohumeral joint and shape of muscle footprints on the scapula have also changed the lines of action of the rotator cuff and deltoid muscles around the joint. These adaptations have likely resulted in evolutionary changes to the force‐sharing relationship between the rotator cuff and deltoids in humans. As a force couple compressing the humeral head into the glenoid, the modern human rotator cuff is highly reliant on the infraspinatus to counter the superior pull of the deltoids (Howell et al., 1986; Roberts, 1974; Thompson et al., 1996). This dependence emerged in the present computational comparison of the chimpanzee and human. The human model predicted high infraspinatus muscle forces, likely to couple with deltoid superior arm elevation action.

The human stochastic model retained infraspinatus and teres minor dominance, and the resultant cumulative distributions highlighted the high potential for overload of infraspinatus in climbing behaviors and similar high‐load, overhead activities. Primates are subscapularis dominant due to the large degree of axial rotation in the upper extremity during locomotor suspensory climbing and other non‐locomotor hand placement behaviors (Larson & Stern Jr, 1986; Mathewson et al., 2014; Stern Jr & Larson, 2001). Similarly, the primate supraspinatus provides synergistic stability over a large range of motion and abducts the arm into overhead postures only assumed in climbing primates (Mathewson et al., 2014; Tuttle & Basmajian, 1978). However, the modern human glenohumeral joint is more reliant on the infraspinatus muscle than other primates to provide axial rotation, adduction, and stability (Mathewson et al., 2014). As humans devolved locomotor usage of their upper extremity, the shoulder form reorganized toward non‐weight‐bearing, below shoulder‐height behaviors (Bramble & Lieberman, 2004; Roach et al., 2013). The human subscapularis and supraspinatus engage in fewer high force tasks, negating large force‐producing capabilities in these muscles (Potau et al., 2007). Both muscles remain important synergists in modern behaviors such as push and pull tasks and below‐shoulder‐height axial rotation tasks (Lee et al., 2000; McDonald et al., 2012). As a result, this has placed more responsibility on the human infraspinatus muscle in overhead tasks, making it susceptible to overload and fatigue. Infraspinatus fatigue initiates a sequence of biomechanical events that may lead to subacromial impingement and rotator cuff injury in humans (Borstad et al., 2009; Ebaugh et al., 2006). Reliance on infraspinatus, despite variation of evolutionarily significant musculoskeletal features, highlights the low efficacy of the modern human glenohumeral joint in overhead, weight‐bearing activities, and unique propensity for rotator cuff disease.

The complexity of the form–function relationship at the scapula, as it relates to human evolution and the last common ancestor, was highlighted by this work. Sensitivity factors indicated that muscle attachment positions have the greatest effect on muscle force distributions. Yet, this was not a direct relationship, as perturbation of each muscle attachment position affected numerous output muscle distributions. Species‐specific variability of all musculoskeletal features biomechanically interacts to support joint function. Specific morphological traits can be associated with specific species, taxa, physical behaviors, and locomotion. However, based on the present results, caution should be used when attempting to isolate specific boney features as being indicative of the hominin musculoskeletal phenotype. Previous analysis has highlighted the diverse interpretation of scapular shape in brachiating primates (Green et al., 2016; Lovejoy et al., 2009; White et al., 2015; Young et al., 2015). The effect of specific features on function, particularly specific muscle positions, is diffused, affecting physical capacity in numerous ways (Stern & Susman, 1991). Subsequently, the relationship between morphological traits and functional capacity needs to be carefully considered. While the present analysis was limited in the scope of probabilistically modeled musculoskeletal features, those that were modified have received significant attention in the literature and have been linked to arborealism, human evolution, and the last common ancestor. The combined perturbation of these features did not result in a more arboreally adapted human glenohumeral joint. Therefore, the current human scapula and scapulae with strong anatomical similarities to the modern human do not constitute an arboreal bone. Rather, either larger perturbation ranges of the current features are needed, or more features—possibly including those on other bones of the shoulder girdle—must also be concurrently adapted to induce arboreal capacity at the human shoulder. Both of these outcomes would suggest that much larger musculoskeletal adaptations are necessary for the human shoulder to increase capacity for arborealism. Continuing research on human evolution and the last common ancestor should take into account what traits are correlated and what traits should be considered in combination to design an arboreal shoulder.

### 4.4 Limitations

There are limitations to using probabilistic modeling to infer biological phenomena. First, the probabilistic model depends on the biological realism of the deterministic model. The chimpanzee model was purposely designed to parallel the preexisting human model for direct comparisons between species (MacLean & Dickerson, 2020). Computational decisions in both models, such as physiological cross‐sectional area of each muscle, simplification of muscle architecture, and attachments as single points, static analysis, and the selection of optimization routine objective function for resolving muscle forces are among the choices that may affect the biological realism of the muscle force outputs of both models. While the differences between the two species in each model may not perfectly reflect biological reality, due to the similar structure of both models, the differences in muscle force outputs do reflect computational biomechanical differences. These results can be used to infer differences in biomechanical function. Second, probabilistic modeling is dependent on the input and output variables. The applied hand forces in both models were very conservative and likely led to lower muscle force predictions than what would be experimentally observed. Given that some human muscles were predicted to be recruited maximally, this only emphasized the human adaptation away from weight‐bearing, repetitive, overhead behaviors. Only a select few evolutionarily derived inputs were considered. Numerous other glenohumeral musculoskeletal features have been associated with function (Larson, 1995; Larson & Stern Jr, 1986; Mathewson et al., 2014; Voisin et al., 2014). Inclusion of these inputs may prompt more overlap between species in muscle force distributions. This is especially true of features that are strongly, mutually associated, such as glenoid inclination and scapular spine angle. Further, only rotator cuff muscle distributions were analyzed in the probabilistic analysis. It is possible that the inputs influenced the behaviors of other muscles not considered, such as the deltoids, and other geometric features such as subacromial space. Future work should consider the analysis of other evolutionarily relevant shoulder muscle groups. Finally, muscle attachment sites were shifted without considering the possibility of concurrent effects of specific directional shifts on the muscle size.

The lack of effect of some input variables may be due to the variation used or not including highly associated musculoskeletal features in the probabilistic analysis. Moderate standard deviations were applied to all input variables to mimic proposed evolutionary shifts in bone shape that could affect glenohumeral biomechanics. Some of variations were less than those used in a previous probabilistic analysis, such as stability ratios (Chopp‐Hurley, 2015). Changes made to some geometric features, such as glenoid inclination, may require concurrent changes not addressed here to generate the hypothesized effect. Based on the present results, physical features being perturbed in isolation of other highly correlated physical features likely do not substantially modify functional performance.

Monte Carlo analyses output sensitivity factors that must be interpreted with some caution. First, sensitivity factors produced by Monte Carlo are not presented in normalized space that allows cross‐comparison of each of the inputs, but as the effect of shifting an input factor by its single standard deviation unit on the output distribution. While magnitudes can only be qualitatively compared across numerous inputs of different variability, the most significant inputs for all outputs were those with the smallest standard deviation unit (muscle insertions). Second, while sensitivity of the output distributions is demonstrated in the factors, the actual directional effect of each input on the output is undefined. It may be worthwhile in future work to assess the sensitivity of output values by performing isolated, standardized perturbations in each of the inputs to determine the magnitude and direction of the effect of each input, as these could have direct pathomechanical or evolutionary consequences. Finally, due to averaging, there is a loss of resolution of the sensitivity factors with respect to probability levels and climbing static instances. Sensitivity values were averaged to consolidate and improve generalizability of the results. Averaging sensitivity factors is a common procedure (Laz & Browne, 2010). Close inspection of the sensitivity factors determined limited changes to sensitivity factors across probability levels and arm suspension static instances, likely mitigating this concern.

## 5 CONCLUSION

Differences between human and chimpanzee rotator cuff function persisted in the probabilistic simulation. Humans remained reliant on the infraspinatus and teres minor and were predicted to overload both muscles in the support phase of a climbing behavior. Chimpanzees had more dispersed muscle forces across all rotator cuff muscles for the horizontal bimanual arm suspension support phase. These outcomes confirm a probable evolutionary foundation of rotator cuff injuries in modern humans. The complexity of the form–function relationship has also been highlighted by this work. Sensitivity factors indicated that muscle attachment positions are most influential in modulating muscle force distributions and affected numerous output muscle distributions. While physical form clearly affects function, the interdependency of the glenohumeral joint complicates isolation of individual features as limits for a specific ability. The species‐specific variability of all musculoskeletal features biomechanically interacts to support joint function.

Computational and probabilistic modeling is increasingly used in a variety of scientific fields but is still novel in evolutionary science. Computational modeling can build upon previous comparative work by integrating the entire musculoskeletal system into an evolutionary and biomechanical analysis of human function in a variety of scenarios (Hutchinson, 2012; O'Neill et al., 2013). Incorporating probabilistic methods increases the faculty of computational models to evaluate the effect of specific physical features on population level function (Laz & Browne, 2010).

## FUNDING INFORMATION

This research was partially funded with combined support from a Natural Sciences and Engineering Research Council (NSERC) of Canada Discovery Grant (311895–2016) and a Canada Research Chairs grant in Shoulder Mechanics.

## CONFLICT OF INTEREST STATEMENT

The authors have no competing interests to declare.

## Data Availability

Data sharing is not applicable to this article as no new dataset was created. Data was probabilistically and randomly derived through computational models.
